# Comparing isoflurane and desflurane: A prospective randomised blinded clinical trial in horses undergoing elective surgery

**DOI:** 10.1002/vetr.70225

**Published:** 2025-12-30

**Authors:** Kate White, John Hird, Polly Taylor

**Affiliations:** ^1^ School of Veterinary Medicine and Science University of Nottingham Nottingham UK; ^2^ Hird and Partners Shelf Equine Hospital Halifax UK; ^3^ Taylor Monroe Ely UK

## Abstract

**Background:**

The recovery period is associated with the greatest risk of injury for horses undergoing anaesthesia. Recovery quality and duration can be influenced by the volatile agent.

**Methods:**

This prospective, randomised blinded clinical investigation recruited 101 healthy client‐owned horses undergoing elective surgery at one UK equine hospital. Anaesthesia was standardised, and horses were randomly assigned to receive desflurane or isoflurane for maintenance. Horses were ventilated to normocapnia and received dobutamine to maintain mean arterial blood pressure above 60 mmHg. All the patients received romifidine intravenously prior to recovery, which was timed and video recorded to allow offline blinded evaluation by two experienced clinicians.

**Results:**

There was no significant difference between groups in haemodynamic support during anaesthesia or recovery quality. Desflurane horses recovered faster and made fewer attempts to stand.

**Limitations:**

Nonlinearity and lack of validation of the recovery scale limit reliability; however, in the absence of a validated instrument, this scoring system is widely used.

**Conclusion:**

Haemodynamic support required during anaesthesia and recovery quality were similar between agents. Desflurane horses recovered faster and required fewer attempts to stand, suggesting that this anaesthetic may lead to fewer recovery injuries and optimise workflow in the equine theatre.

## INTRODUCTION

The recovery period is associated with the greatest risk of injury for horses undergoing anaesthesia. Volatile anaesthetics that undergo minimal metabolic breakdown and are rapidly eliminated may improve the quality of recovery from anaesthesia in horses.^1^ Desflurane has a lower blood‒gas partition coefficient (0.57) than isoflurane (1.45) and sevoflurane (0.74) and should theoretically provide a faster return of consciousness.^2^ However, studies investigating recovery quality with different volatile agents have indicated that although the recoveries may be quicker^3^, ^4^ they do not guarantee calm coordination.^5^, ^6^ Furthermore, the recovery aspects of studies are challenging to interpret, as different recovery scoring tools are employed.^7^ Many laboratory studies use both small numbers of horses and induction via facemask delivery^3^, ^8^, ^9^ in contrast to premedication and intravenous (i.v.) induction differences highly likely to influence the recovery profile.^5^

Clarke et al. studies evaluated recovery quality following desflurane and utilised xylazine‒ketamine induction and xylazine sedation in recovery; recovery quality was rated as excellent and good^10^, ^11^; excellent recovery was similarly reported following ketamine‒medetomidine induction and desflurane with medetomidine infusion for maintenance.^12^ A retrospective study comparing recovery after isoflurane or desflurane for equine ‘tie‐back’ procedures found little difference between the volatile agents for quality and attempts to stand but a significantly shorter recovery time for desflurane.^4^

Therefore, our aim was to compare isoflurane and desflurane anaesthesia in horses undergoing elective surgery; the primary outcome measure was recovery quality. There is sufficient evidence and consensus for using an alpha 2 agonist to improve equine recovery,^13^, ^14^ so romifidine was administered to all horses at the end of the procedure.

## MATERIALS AND METHODS

### Study design

A prospective, randomised, blinded, controlled clinical trial was conducted in a UK equine hospital under an Animal Test Certificate (ATC‐S‐152) and ethics committee approval (University of Nottingham, School of Veterinary Medicine and Science 3327210205). Informed client consent was obtained, and horses were randomly allocated for anaesthesia maintenance with either isoflurane (I) or desflurane (D) (GraphPad QuickCalcs 2019). Recovery from anaesthesia was recorded on video and two experienced ECVAA diplomates unaware of which volatile agent had been administered graded each recovery.

### Animals

The sample size calculation (G*power 3) indicated that 46 horses per group would be required to show a statistically significant and clinically relevant difference. Based on pilot studies using a 1–5 simple descriptive scale for recovery quality,^15^ for 80% power and 95% confidence of detecting a 33% difference in recovery scores, it was estimated that the sample size should be 98 horses. An ordinal logistic regression model relying on a proportional odds assumption was used. An additional 10 above the required 92 was used to allow for case exclusion or data collection problems. The inclusion criteria were defined as elective surgical cases in horses or ponies older than 6 months that had not received sedation in the preceding 24 hours. All horses had access to hay and water in their stables. Patients were weighed and underwent a full clinical examination prior to premedication. All were classified as ASA 1 under the American Society of Anaesthesiologists classification.

### Premedication

Acepromazine (0.03 mg/kg; Neurotranq, Alfasan) and flunixin (1.1 mg/kg; Pyroflam, Norbrook) were injected i.v. at least 30 minutes prior to anaesthesia. A 12‐gauge over the needle cannula (Intraflow, Vygon) was aseptically placed in the jugular vein following subcutaneous injection of lidocaine (1 mL; Lignol, Dechra). Perioperative antibiotics were determined by the clinician in charge of the case and consisted of 12 mg/kg procaine benzylpenicillin (Depocillin, MSD) intramuscularly with or without 6.6 mg/kg gentamicin (Genta‐Equine, Dechra) or 5 mg/kg oxytetracycline (Engemycin, MSD) i.v. Ten minutes before induction, 80 µg/kg romifidine (Sedivet, Boehringer Ingelheim) and 0.2 mg/kg morphine (Morphine sulphate, Martindale Pharmaceuticals) were administered i.v., and the horse was left undisturbed.

### Anaesthesia induction and maintenance

Anaesthesia was induced with a combination of i.v. midazolam (0.06 mg/kg; Dormazolam, Dechra) and ketamine (3 mg/kg; Ketavet, Zoetis), and horses became recumbent without manual restraint. The trachea was intubated with an appropriately sized cuffed, silicone endotracheal tube and the horse was hoisted onto a padded mattress of a large animal surgery table and positioned in dorsal or lateral recumbency, depending on the procedure. The endotracheal tube was connected to a large animal anaesthetic machine (JD LAVC 2000, JD Medical) and horses were mechanically ventilated (Bird Mark 7, JD Medical) with a positive inspiratory pressure of 15–20 cmH_2_O, a tidal volume of 10−12 mL/kg and a respiratory rate (RR) selected to maintain end tidal carbon dioxide pressures (PE′CO_2_) of 40−45 mmHg (5.3−6.0 kPa). Anaesthesia was maintained with isoflurane (Isoflo, Zoetis) or desflurane (Desflurane, Piramal) in oxygen. Vaporiser settings were selected to maintain end tidal isoflurane (FE′ISO) between 1% and 1.5% and end tidal desflurane (FE′DES) between 7.5% and 8.5%. Waste desflurane was captured into charcoal cannisters (AGA, Burtons), and waste isoflurane was vented through the active gas scavenging system. Lactated Ringer's solution (Vetivex [Hartmann's] 11, Dechra) was infused i.v. at 5 mL/kg/h for the first hour and 2 mL/kg/h thereafter. Dobutamine solution (Dobutamine concentrate, Hamelm Pharmaceuticals) was infused i.v. (HK‐100VET infusion pump, VETisco) to maintain mean arterial blood pressure (MAP) above 60 mmHg, starting at 0.25 µg/kg/min to a maximum of 2 µg/kg/min. The total amount of dobutamine administered per horse was recorded. A urinary catheter was placed in all horses before surgery started. Locoregional anaesthesia was at the discretion of the surgeon and performed only in horses undergoing castration, in which testicular infiltration was administered using 5–15 mL of lidocaine per testicle. If the horse developed nystagmus or moved, a ketamine or thiopentone i.v. bolus (at the discretion of the anaesthetist) was administered to improve the plane of anaesthesia.

### Monitoring

A 22 or 20‐gauge catheter (Intraflow, Vygon) was placed in a facial or dorsal metatarsal artery for arterial blood pressure measurement. Heart rate (HR); systolic, mean and diastolic blood pressure; RR; and arterial oxygen haemoglobin saturation (SPO_2_), PE′CO_2_, FE′ISO or FE′DES were continuously displayed using a multiparameter monitor (Carescape B850, GE) and manually recorded every 5 minutes. Post procedure, the hypotensive index (mmHg below a pre‐determined value^16^) for MAPs of 60 and 70 mmHg was calculated to quantify the magnitude and duration of hypotension. Monitoring devices were calibrated prior to use according to the manufacturer's instructions.

### Anaesthesia recovery

At the end of surgery horses were moved into a padded recovery box. Ventilation was assisted using a demand valve (Oxygen Demand Valve, JD Medical) in apnoeic horses until spontaneous respiration resumed. Once spontaneous ventilation was deemed satisfactory by the attending anaesthetist, 20 µg/kg romifidine was administered i.v., a nasal tube was placed, the endotracheal tube was removed and the horse was left to recover unassisted. The entire recovery phase was timed and scored by the attending anaesthetist and filmed by a camera (Dahua IP Camera system) situated above the recovery box. Films (with audio) were saved as MP4 files and stored securely for offline analysis by two anaesthetists (K.W. and P.T.) unaware of the volatile agent used. The times from disconnection to sternal recumbency and standing were recorded. The quality of the anaesthesia recovery was evaluated using a previously reported five‐point recovery score system for horses^15^, ^16^ (5 best‒1 worst, Table 1). The number of attempts to stand was counted, and a free text box was filled with a narrative account of the recovery to translate the descriptors into a ‘word cloud’ (www.WordClouds.co.uk) to improve future recovery scoring. The authors discussed and agreed on the application of the scoring system, and several ‘practice runs’ were undertaken on clinical cases anaesthetised before the trial began to ensure consistency.

**TABLE 1:** Recovery scoring system.

Score	Description
5	No ataxia, no struggling, stood up at first attempt as if fully conscious
4	Slight ataxia and struggling, stood up at first/second attempt, no serious instability
3	Excitement, paddling when recumbent, several attempts to stand, severe ataxia once standing, may fall, danger of self‐inflicted injury
2	Excitement when recumbent, persistent unsuccessful attempts to stand, severe ataxia when standing, aimless walking and high risk of self‐inflicted injury
1	Very violent (‘wall of death’) self‐inflicted injury, prolonged struggling or unable to stand 2 hours after the end of anaesthesia

*Note*: The number of attempts to stand was also counted, and a free text box was used for a narrative account of the recovery by the scorer.

### Data analysis

Data normality was evaluated using the Shapiro‒Wilk test. Categorical data were analysed using Fisher's exact test, and kappa was calculated for inter‐rater reliability. Continuous data and physiological variables were analysed using the unpaired Student's *t*‐test for normally distributed data or the Mann‒Whitney test for nonparametric data. The correlation between the subjective recovery score and other relevant variables was tested by calculating the Spearman rank correlation coefficient and a multivariate mixed effects model was used to analyse the data set with the volatile agents as the fixed between subjects factor (GraphPad Prism 10.4.2). Differences were considered significant when *p‐*value is 0.05 or less.

## RESULTS

One hundred and one horses were recruited to the study between December 2021 and June 2022. Two horses were excluded because the recovery film was not available. The final analysis included 51 horses who received desflurane and 48 who received isoflurane. No horses underwent multiple anaesthetics.

There were no significant differences between the groups for age, bodyweight, sex, duration of surgery, duration of anaesthesia, type of surgery, position of horse during anaesthesia or antibiotic administration. The same surgeon performed all the procedures. The patient characteristics are summarised in Table 2.

**TABLE 2:** Demographic data, procedural times and types of surgery of horses undergoing anaesthesia maintained with isoflurane (I, *n* = 48) or desflurane (D, *n* = 51).

	I (*n* = 48)	D (*n* = 51)	95% CI	*p*‐value
Age	8.3 ± 3.8	8 ± 3.9	‒1.3 to 1.8	0.7
Sex				
Female	20	19		
Male	29	32		
Castrated	27	28		
Stallions/colts	2	4		
Breed				N/A
Pony (Connemara, Welsh)	5	4		
Cob	5	4		
Thoroughbred and thoroughbred x	7	2		
Warmblood (Hannovarian, Holsteiner, Sport Horse)	23	27		
Irish Sport Horse	5	4		
Irish Draft Horse, Clydesdale	4	6		
Arab	2	3		
Unspecified	0	1		
Weight (kg)	543 ± 60	548 ± 96	−37.8 to 26	0.7
Duration of anaesthesia (minutes)	83 ± 23	86 ± 27	−12.4 to 7.5	0.6
Duration of surgery (minutes)	40 (15–95)	40 (15–120)	−10 to 5	0.8
Types of surgery				N/A
Arthroscopy/tenoscopy/bursoscopy (1)	12^a^	11		
Arthroscopy/tenoscopy/bursoscopy (>1)	12	12^a^		
Neurectomy	14	18		
Annular ligament desmotomy	4^a^	2^a^		
Splint bone removal	0	2		
Airway surgery	0	1		
Sarcoid removal	2	3		
Castration and cryptorchid surgery	1	2		
Wounds and abscess debridement	3	1		
Antibiotic administration				
Perioperative penicillin	31	43		
Perioperative penicillin and gentamicin	14	8		
Perioperative oxytetracycline	3	0		
No perioperative antibiosis	0	0		
Recumbency				
Dorsal	39^b^	38		
Right lateral	7	8^c^		
Left lateral	2	4		
Unspecified	0	1		

*Note*: Normally distributed data are presented as mean ± standard deviation and nonparametric data as median (minimum‒maximum). The *p*‐value was calculated for continuous data using Student's *t* test or the Mann‒Whitney test, categorical data were compared using Fisher's exact test or the chi‐squared test. The *p*‐value represents differences between groups, and confidence intervals (CIs) are calculated for that difference.

^a^ One horse had an additional procedure.

^b^ One horse in the I group was moved from dorsal to left lateral recumbency during anaesthesia.

^c^ One horse in the D group was moved from right to left lateral recumbency during anaesthesia.

The minimum alveolar concentration (MAC) multiples were calculated for each horse as averaged end tidal volatile agent divided by the MAC of the volatile agent^17^ and there was no difference between groups. Total anaesthetic exposure calculated as MAC hours were similar in the two groups (Table 3). MAC multiples calculated at the point of disconnection were significantly (*p* = 0.0009) greater in D horses (1.08) compared to I horses (1.04).

**TABLE 3:** Anaesthetic data for the two groups of horses.

	Isoflurane	Desflurane	95% CI	*p*‐value
Dobutamine dose (µg/kg/min) infused during anaesthesia	0.42 (0.21‒1.5), *n* = 39	0.5 (0.11–1.2), *n* = 48	‒0.15 to 0.04	0.28
Duration of dobutamine infusion (minutes)	50 (5‒95)	60 (15‒135)	‒20 to 5	0.19
Additional intraoperative ketamine (mg/kg)	0.58 (0.4‒0.76), *n* = 2	0.8 (0.4‒1.6), *n* = 5^a^	‒0.21 to 0.47	0.44
Additional intraoperative thiopentone (mg/kg)	0	1.0 (0.8‒2.7), *n* = 4^a^	‒0.11 to 0.61	0.12
Average vaporiser setting (%)	2.4	9.5	N/A	N/A
End tidal volatile agent (%)	1.39 (1.2‒1.6), *n* = 48	7.8 (6.1–8.8), *n* = 51	N/A	N/A
MAC multiples	1.07 (0.9‒1.2), *n* = 48	1.06 (0.9‒1.2), *n* = 51	‒0.04 to 0.013	0.38
MAC multiple at disconnection	1.04 (0.9–1.2), *n* = 48	1.08 (0.9–1.3), *n* = 51	0.018 to 0.081	0.0009
MAC hours	0.8 (0.5‒1.8), *n* = 48	0.8 (0.4‒1.8), *n* = 51	‒0.09 to 0.07	0.84
Supplemental oxygen and ventilation via demand valve in recovery	*n* = 20	*n* = 16	‒0.083 to 0.34	0.3

*Note*: Values are expressed as mean ± standard deviation or median (minimum‒maximum). *p*‐value represents differences between groups, and confidence intervals (CIs) are calculated for that difference. MAC multiples were calculated as end tidal volatile agent divided by isoflurane MAC of 1.3 vol% or a desflurane MAC of 7.4 vol%. MAC hour was calculated as (average end‐expired volatile agent concentration/MAC _Volatile agent_) × (hours of volatile agent anaesthesia), based on an isoflurane MAC of 1.3 vol% and a desflurane MAC of 7.4 vol%.

Abbreviation: MAC, minimum alveolar concentration.

^a^ One horse received thiopentone and ketamine top ups.

There was no significant difference (*p* = 0.06) in the number of boluses of thiopentone (*p* = 0.12) or ketamine (*p* = 0.44) required in either group to improve the plane of anaesthesia. Excluding the ‘topped up’ horses made no difference (*p* = 0.41) to the overall recovery scores. There was no significant difference between the groups for the number of horses receiving dobutamine (*p* = 0.06), the total dose infused (*p* = 0.28) or the duration of infusion (*p* = 0.19). The I group SpO_2_% was significantly greater than that of the D group (*p* = 0.02) (Table 4).

**TABLE 4:** Cardiopulmonary data for the two groups of horses, anaesthetised with isoflurane or desflurane.

	Isoflurane	Desflurane	95% CI	*p*‐value
Heart rate (beats per minute)	36 (29–56)	34 (22–54)	−2.5 to 1.4	0.5
PE′ CO_2_ (mmHg)	40 (35–60)	40 (32–46)	−0.8 to 0.9	0.8
SpO_2_ (%)	99 (93–100)	98 (90–100)	0.016 to 1.09	0.02
Hypotensive index 60 (mmHg/h)^a^	3.5 (0.17–14), *n* = 32	3.5 (0.05–13.3), *n* = 43	−1 to 2.3	0.4
Hypotensive index 70 (mmHg/h)^b^	14.15 (0.08–40.3), *n* = 44	13.2 (2.3‐47.9), *n* = 48	−3.4 to 3.1	0.9
Mean arterial pressure (mmHg)	63 (55–82)	62 (54–81)	−3.9 to 1.2	0.3

*Note*: Nonparametric data are reported as median (minimum‒maximum). *p*‐value represents differences between groups, and confidence intervals (CIs) are calculated for that difference.

^a^ Hypotensive index = (60 – lowest mean blood pressure) × time in hours when the mean blood pressure was less than 60.

^b^ Hypotensive index = (70 – lowest mean blood pressure) × time in hours when the mean blood pressure was less than 70.

There was a moderate positive correlation (0.4) between Hypo 70 index and recovery time in the D horses (*p* = 0.023) and a significant negative correlation for recovery time and MAP (‒0.34) and HR (‒0.4; *p* = 0.012) (i.e., D horses with a lower HR and blood pressure were likely to take longer to stand).

Horses in the isoflurane group attained sternal recumbency significantly later than the desflurane group (*p* < 0.0001), and the time to standing was significantly shorter for the desflurane group (*p* < 0.0001) (Table 5).

**TABLE 5:** Recovery characteristics and recovery scores assigned by attending anaesthetist and two blinded observers of all horses after isoflurane or desflurane anaesthesia and sedation with romifidine 20 µg/kg intravenously.

	Isoflurane (*n* = 48)	Desflurane (*n* = 51)	95% CI	*p*‐value
Attempts to stand	4 (1‒12)	2 (1–9)	‒2 to 0	0.08
Time to sternal (minutes)	30 (6‒47)	21 (7–46)	‒11 to ‐4	0.0001
Time to standing (minutes)	41 (27‒74)	30 (10‒67)	8 to 17	0.0001
Recovery score				
Attending anaesthetist	4 (1‒5)	4 (3‒5)	‒1 to 0	0.09
Blinded observer (K.W.)	4 (1‒5)	4 (2‒5)	‒1 to 0	0.25
Blinded observer (P.T.)	3 (1‒5)	4 (2‒5)	‒1 to 0	0.58

*Note*: These nonparametric data are presented as median (minimum‒maximum). *p*‐value was calculated for continuous data using Mann‒Whitney test. *p*‐value represents differences between groups, and confidence intervals (CIs) are calculated for that difference.

The Spearman's correlation coefficient between the two blinded observers was 0.84 (confidence interval 0.76‒0.89; *p* < 0.0001); the overall *k*‐weighted value was 0.68. When horses were divided into D and I groups, the Spearman's correlation coefficient between the two blinded observers for I was 0.86 (confidence interval 0.75‒0.92; *p* < 0.0001); the overall *k*‐weighted value was 0.73. The Spearman's correlation coefficient between the two blinded observers for D horses was 0.81 (confidence interval 0.68‒0.89; *p* < 0.0001); overall *k*‐weighted was 0.56. The scores are summarised in Table 5. There were no differences in recovery score between D and I horses when evaluated by either the blinded scorers or the attending anaesthetist aware of the volatile agent identity.

The recovery score and the number of attempts to stand were negatively correlated for both volatile agents. The Spearman's correlation coefficient was ‒0.91 (confidence interval ‒0.95 to ‒0.85; *p* < 0.0001) for desflurane and ‒0.39 (confidence interval ‒0.62 to ‒0.11; *p* < 0.0063) for isoflurane.

**FIGURE 1:**
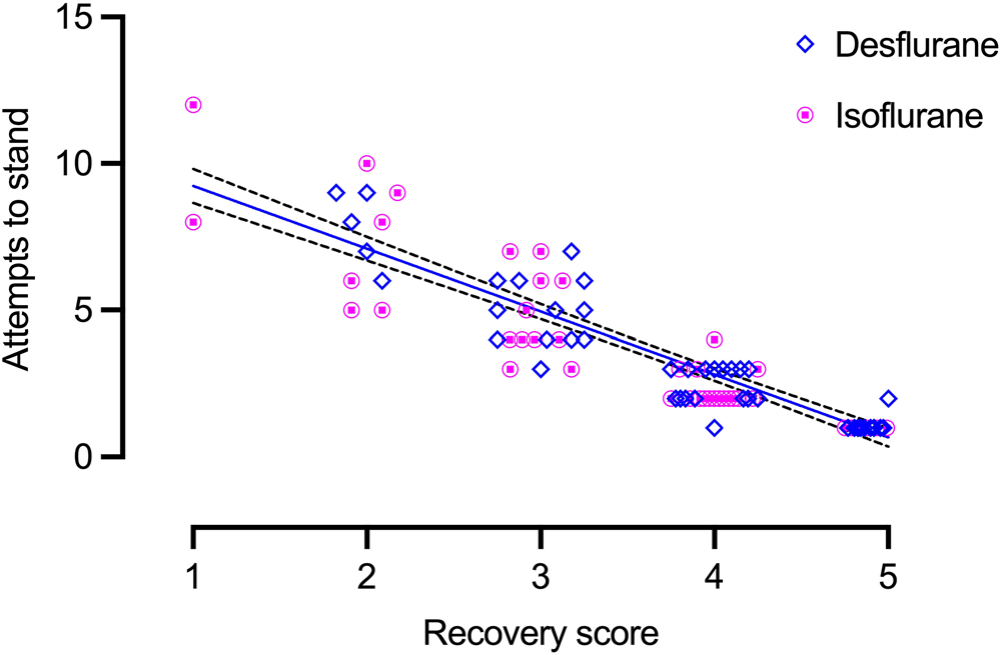
Scatterplot and linear regression line (solid line) with 95% confidence bands (dashed line) of horses’ attempts to stand and the overall recovery score.

Linear regression plots of the recovery scores and attempts to stand were not significantly different between D and I horses, and pooled data from both groups are represented in Figure 1.

Five horses in the D group had a score of 1 or 2, and seven in the I group. The patient characteristics, surgical procedure, duration and volatile agent are summarised in Table 6. Sixty percent (6/10) of the horses with a recovery score of 2 had undergone neurectomy.

**TABLE 6:** Patient and procedure details of the poor recoveries (scores 1 and 2) anaesthetised with isoflurane (I) or desflurane (D).

	Surgical procedure	Volatile agent	Position, GA duration, surgery duration (minutes)	Patient weight (kg), age (years), breed and sex
Recovery score 1	Ventriculocordectomy and arytenoid lateralisation HL annular ligament desmotomy (1)	I I	RL 105 (70) RL 102 (40)	632 kg 8Y Shire X G 554 kg 12Y Welsh Section D F
Recovery score 2	Arthroscopy (1) Splint bone removal (1) and HL N&F (2) Arthroscopy (2) Arthroscopy (2) and HL N&F (2) Penile tumour removal HL N&F (2) HL N&F (2) HL N&F (2) Palmar digital neurectomy (2)	D D D D D I I I I I	D 55 (20) LL 80 (30) D 85 (35) D 95 (30) D 105 (60) D 140 (95) D 75 (40) D 75 (40) D 90 (40) D 95 (45)	774 kg 11Y WB G 618 kg 5Y WB G^a^ 433 kg 11Y Fr trotter G 544 kg 6Y WB G 596 kg 6Y WB F 526 kg 10Y WB G^b^ 616 kg 6Y IDH G 500 kg 8Y TB G 534 kg 6Y WB F 508 kg 12Y ISH F

Abbreviations: D, dorsal recumbency; Fr, trotter French Trotter; G, gelding; GA, general anaesthetic; HL, hindlimb; IDH, Irish Draft Horse; ISH, Irish Sports Horse; RL, right lateral recumbency; LL, left lateral recumbency; TB, thoroughbred; Shire X, Shire Horse cross; WB, warmblood; N&F, suspensory ligament neurectomy and fasciotomy; Welsh Cob D, Welsh Section D Cob.

^a^ Fifteen minutes before end one bolus of thiopentone (500 mg) was administered intravenously to improve the plane of anaesthesia.

^b^ Haemorrhage was identified as the horse arrived in the recovery box on the winch necessitating immediate return to theatre to address the surgical site bleeding, requiring 400 mg ketamine intravenously. This emergency intervention lasted a further 15 minutes. Total blood loss was considered clinically insignificant. Anaesthesia and surgical time include the emergency intervention.

## DISCUSSION

Fatal or career‐ending injuries sustained during recovery from anaesthesia in horses remain a welfare and financial concern for the veterinary team and owners. The ambition to mitigate these catastrophes by utilising different drug protocols is therefore commendable. This prospective clinical study revealed few clinical differences attributable to the choice of volatile agent. The primary outcome measure was the overall recovery score, and this was similar in the D and I horses. There was no significant difference in the duration of surgery or anaesthesia of the sub optimal recoveries. However, the poorer recoveries (score 2) included a disproportionate number of neurectomy procedures, all undertaken without locoregional analgesia; it is possible that pain contributed to the suboptimal recovery quality.

There was no significant difference between groups in cardiorespiratory function, but horses with lower blood pressure and HR appeared to take longer to stand. The relationship between hypotension and post operative myopathy remains an important concern for equine anaesthetists.^18^, ^19^ Notwithstanding the multitude of factors affecting recovery, those within the remit of control by the attending anaesthetist, such as blood pressure, must be prioritised. There was a significant difference, albeit nonclinical, in the SPO_2_% between groups; however, these values may be unreliable in view of many missing values and need for frequent repositioning of the probe to generate a reading. Serial blood gases were not indicated in these elective cases; therefore, it was not possible to correlate the incidence of relative hypoxaemia. Clinically, the differences had minimal impact on the horses with all SPO_2_% above 90%.

Our findings reinforce the view that time to stand is influenced by multiple factors and does not correlate well with quality of recovery, which is, after all, the most pertinent outcome measure. Longer duration of anaesthesia has been shown to influence recovery quality negatively in halothane and isoflurane studies,^15^, ^20^, ^21^, ^22^ but comparable information about desflurane is lacking. The horse's total body drug uptake as ratio of drug potency is lower for desflurane compared to equipotent doses of other volatile agents and these differences increase with time^1^, ^2^ offering potential advantages of desflurane. From a pharmacokinetic standpoint, it could be inferred that desflurane's profile best suits it for facilitating a rapid recovery in large horses after a prolonged anaesthetic.^1^ In our study, the MAC multiples across the anaesthetic were comparable, but this surrogate marker of plane or depth of anaesthesia for D horses at disconnection was significantly greater than for I horses. It is unclear whether this difference in residual desflurane and isoflurane in the horses’ tissues affected recovery scores but may explain why fewer I horses required intraoperative top ups of ketamine or thiopentone. Isoflurane has a higher blood gas solubility than desflurane meaning more volatile agent accumulates in muscle, splanchnic organs and especially fat, which act as an isoflurane reservoir up until the anaesthetic partial pressure in the tissue equilibrates with that in arterial blood. One model of anaesthetic uptake and distribution validated in people confirmed that volatile agent remains in the body for days after recovery; after 3 hours of isoflurane, it takes 71 hours to eliminate 99.9% from the brain, although 8% of the administered dose would remain in the body.^2^ The differential in washout kinetics^23^ between both agents supports our findings of a more rapid recovery using the less soluble desflurane, and the subjective observation of improved coordination in the D horses could be inferred.

The indexes using MAC in this study need to be interpreted with caution since the outputs can be altered by the MAC value chosen from the source material, and the population variability associated with this parameter. For example, mean MAC values of 7.4%‒8.06% are quoted for desflurane^8^, ^24^, ^25^ and 1.31%‒1.64% for isoflurane^26^, ^27^ with lower values in ponies.^28^ Notwithstanding this limitation, the MAC indexes indicate comparable exposure to volatile agent between D and I groups.

A number of horses received boluses of ketamine or thiopentone intraoperatively with one D horse receiving both drugs. The small numbers preclude meaningful interpretation of whether these drugs affected recovery scores. Infusions of ketamine have been shown to affect recovery quality or recovery time in horses sedated with α2‐adrenoreceptor agonists^29^; thus, administration of the ketamine or thiopentone boluses intraoperatively could potentially could have altered the recovery in our horses. Two that received boluses within 15 minutes of recovery had a poor recovery (score 2). One horse that underwent a penile tumour removal was returned to theatre after bleeding was noticed in recovery and received 0.75 mg/kg ketamine to facilitate transport back to theatre. The second horse received a bolus of thiopentone 15 minutes prior to recovery. Other studies evaluating recovery have excluded animals receiving ‘top ups’ within 30 minutes of recovery^30^; in our study, excluding the ‘topped up’ horses made no difference to the overall recovery scores.

Our horses were disconnected from the ventilator and transferred to a recovery box adjacent to theatre via a winch and gantry typically taking approximately 60 seconds. Once horses were in recovery, oxygen supplementation using a demand valve was provided in the event of apnoea. There was no significant difference between the D and I groups requiring oxygen supplementation and no correlation between recovery score and the provision of oxygen supplementation. It is hypothesised that additional ventilation in recovery could affect the washout kinetics of the volatile agents and hasten recovery; however, this was not apparent here. Other studies have demonstrated that insufflation of a carbon dioxide mixture during recovery can have a greater effect on recovery than the volatile agent itself.^31^

The D group recovered significantly faster, with fewer attempts to stand. The recovery score and the number of attempts to stand were negatively correlated for both groups. It stands to reason that the more attempts a horse makes, the greater the risk of self‐inflicted injury, adding weight to the view that a better recovery score is likely to be associated with less risk of injury.^32^ A faster recovery may also be beneficial for case throughput and efficient use of theatre space and staff time.

There was substantial agreement between observers for scoring I horses and moderate agreement for D horses. On further evaluation the area of disagreement centred on the difference in opinion in definition of a 4 or 5 score for D horses. The D horses subjectively were swift and coordinated in their movement to stand; however, the scale did not accommodate the sensitivity of an almost perfect recovery following a weak initial futile attempt or a slight stumble. Oftentimes, the horse was stimulated by ambient noise, for example, the mechanical hoist, whinnying of other nearby horses or staff climbing the steps at the side of the recovery box. Controlling for these factors was not possible in the clinical environment. Free text included phrases such as ‘almost perfect’, ‘this was a 4.5!’ or ‘this would have been perfect if not for the initial lurch’. The scoring system has now been refined for future use to accommodate this nuance that was particularly evident in the D group.

There was no significant correlation between time to standing and quality of recovery in either the D or I groups. We hypothesised that recovery quality is likely to be better in a horse that remains in lateral or sternal recumbency throughout the phase during which the central nervous system anaesthetic concentrations are high enough to cause incoordination and ataxia. A longer time to stand has been shown to improve recovery score quality,^15^, ^33^, ^34^ but equally, the opposite has also been demonstrated.^7^, ^35^ Additionally, studies to date have shown that sedation improves recovery quality but prolongs duration.^15^, ^33^, ^36^ One retrospective study found that certain limb and body positions during recovery may be predictive of a successful first attempt to stand, although the complexity of the biomechanical actions of the horse in recovery was acknowledged.^34^

No universal validated instrument to grade recovery quality exists; however, the scoring system used in this study^15^ is widely adopted and considered reliable^37^, ^38^ with high repeatability.^39^ The pictorial representation in the form of word clouds for the recovery scale aims to enhance the scoring of recoveries, the size of words in the clouds reflects their frequency of use by the scorers (Appendix). Additionally, the quantification of the attempts to stand expands the usefulness of the tool (Appendix). Real‐time scoring of recovery by attending anaesthetists is often impractical due to preparation for the next case; recording technology in the recovery box is to be recommended for review of recovery in case it is missed, clinical research studies and clinical governance. Evaluation of the scoring by the reviewers revealed that the audio as well as the visual component to be crucial for comprehensively evaluating recovery and ambient noise.^13^, ^39^

It is essential to consider the impact of the release of desflurane into the environment. Desflurane has a global warming potential 13 times higher than isoflurane,^40^ and reducing its use is an obvious step towards a lower carbon world. Desflurane has been banned in some countries for this reason, and vaporisers have been decommissioned and removed from hospitals despite the benefits the drug offers in some patients. In our study, the desflurane was captured onto charcoal; more recently, the use of an activated carbon VET‐can (SageTech Veterinary) to capture volatile agents for potential re‐use has shown very promising in vivo mass transfer (MT) results in people and small animals.^41^, ^42^, ^43^ In vivo MT is calculated as the difference between volatile capture device weight change (pre‐post procedure) divided by vaporiser weight change (pre‒post procedure). A pilot study has shown 80% in vivo MT is possible for desflurane, significantly higher than that of isoflurane 34% (22%‒54%).^44^ This characteristic is presumably a result of isoflurane being more soluble in the horse's tissues; therefore, less of the delivered isoflurane is available to be captured from the horse. It is likely in the future that a circular economy of use‒capture‒reuse develops to reduce the emissions associated with the release of the volatile anaesthetic agents.^43^ Currently, there is a paucity of data quantifying the environmental impact of the parenterally administered anaesthesia drugs, and it is not possible to make inferences on the carbon footprint of i.v. versus inhaled anaesthetics without detailed life cycle analyses.

There are some limitations to the study to acknowledge. First, pain assessment per se is not an overt component of the recovery scale used and so was not directly assessed, but it is possible that pain could influence the quality of recovery, potentially exacerbated by nociception post‐surgery or low‐dose volatile being pro‐nociceptive.^45^ Human studies of nociception indices have failed to find discrete reliable predictors of acute postoperative pain using surgical pleth index, pupillary pain index and nociception level. However, a degree of accuracy was achieved using a multivariable logistic regression combining patient information, regional analgesia and nociception level values.^46^ The use of locoregional analgesia during anaesthesia has been shown to reduce postoperative mortality in people and in dogs,^47^, ^48^ and opportunities exist for increased use of locoregional techniques in equine anaesthesia; recent data suggest less than 20% of equine anaesthetics incorporate a local anaesthetic block.^14^ The reasons behind the reluctance of any team to use locoregional techniques is worthy of future research, and may include but not be limited to concerns with local anaesthesia toxicity, impaired coordination in recovery, skill and time to perform the block, cost and absence of perceived benefit.

A second limitation was the use of the oxygen demand valve in the recovery box, its use was not standardised, and there is a possibility it affected the washout kinetics of the volatile agent and accelerated the awakening of some patients. Third, the nonlinearity of ordinal scales is an inherent limitation with these types of studies, with scores 4 and 5 being ‘closer’ than a 3 and 4; finally, the lack of validation for this recovery scale limits the reliability and accuracy of the results.

### Future research

The quality of recovery is multifactorial, with some factors being within the control of the anaesthetist and others outwith. To improve recovery quality and reduce premature uncoordinated attempts, future research evaluating additional regional analgesia and partial i.v. anaesthesia protocols is warranted.^14^

## CONCLUSIONS

Required haemodynamic support during anaesthesia and overall recovery quality were similar with desflurane and isoflurane. However, D horses required fewer attempts to stand in the shorter recovery time, suggesting that this anaesthetic may lead to fewer recovery injuries and may help optimise workflow in the equine theatre.

## AUTHOR CONTRIBUTIONS

*Study initiation*: all authors. *Data collection and assimilation*: John Hird. *Assessment of recovery films*: Kate White and Polly Taylor. *Data analysis*: Kate White. *Manuscript writing*: Kate White and Polly Taylor. *Approval of final manuscript*: all authors.

## CONFLICT OF INTEREST STATEMENT

The authors declare they have no conflicts of interest.

## ETHICS STATEMENT

Ethical approval was granted from the University of Nottingham, School of Veterinary Medicine and Science ethics committee 3327210205, and conducted under an Animal Test Certificate (ATC‐S‐152).

## Supporting information



Supporting Information

## Data Availability

The data that support the findings of this study are available from the corresponding author upon reasonable request.
