# Supplementary material for: Comparing isoflurane and desflurane: A prospective randomised blinded clinical trial in horses undergoing elective surgery
Source: Vet Rec. 2025 Dec 30;198(8):e363–72. doi: 10.1002/vetr.70225 (PMC13088929; doi:10.1002/vetr.70225)
Supplement: Supplementary file 1 — Supporting Information [file VETR-198--s001.docx]

5
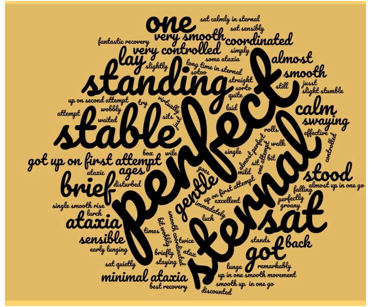

Smooth sensible, coordinated, small lurch, no struggling, stood up at first attempt, little or no ataxia

4
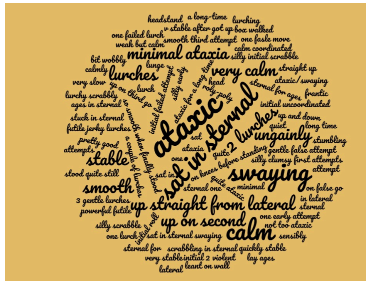

Mild swaying in sternal, minimal struggling, a few attempts to stand (average 3 attempts), no serious instability, slight ataxia once stood

3
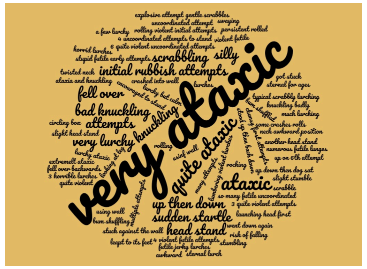

Some scrabbling or rolling, some futility, lurchy, may include moderate swaying in sternal, numerous (average 5 attempts) attempts to stand, may include periods of calm, may show persistent knuckling when stood and some ataxia

2
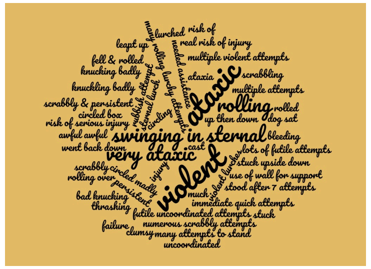

Persistent futile, ‘drunken’ lurches and unsuccessful crashing attempts to stand (average 10 attempts), possibly some excitement, can include multiple rolls and scrabbles, head stands/neck twists.

1
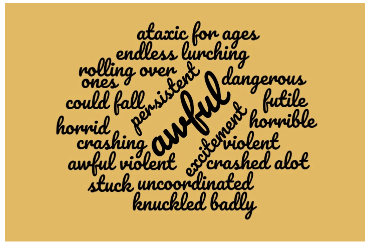

Very determined and violent, self-inflicted injury likely, prolonged futile attempts (average 15 attempts to stand) possibly some excitement, huge lurches, rolling, may require intervention/rescue
